# ADDRESSING THE SUCCESSFUL TRANSITION OF COMMUNITY COLLEGE GERONTOLOGY STUDENTS TO UNIVERSITY SETTINGS

**DOI:** 10.1093/geroni/igz038.900

**Published:** 2019-11-08

**Authors:** Margaret M Manoogian

**Affiliations:** Western Oregon University, Monmouth, Oregon, United States

## Abstract

Developed in 2012, our gerontology program has tracked enrollment, learning outcomes, student competency development, and career paths post-graduation. Enrolled students tend to be older, transferring from community colleges, reentering college due to work and family commitments, and retooling career paths. Our efforts have focused on career integration across all courses culminating in a two-term practicum program. Additionally, through discussions with community college faculty and students, alumni, and community partners, we have adapted our curriculum to address student needs for work, family care, and academic engagement through flexible course delivery, syllabus construction, applied project development, and direct contact with professionals in and outside the classroom. Developing strategies to ensure transfer student success is critical, as well as offering strong career preparation for older students entering the workforce. Comprehensive placement data and an overview of the needs and challenges for university programs to partner with community college programs will be highlighted.

